# Whole blood transcriptome analysis reveals potential competition in metabolic pathways between negative energy balance and response to inflammatory challenge

**DOI:** 10.1038/s41598-017-02391-y

**Published:** 2017-05-24

**Authors:** Juliette Bouvier-Muller, Charlotte Allain, Guillaume Tabouret, Francis Enjalbert, David Portes, Céline Noirot, Rachel Rupp, Gilles Foucras

**Affiliations:** 1 INRA, UMR1388 Génétique, Physiologie et Systèmes d’Elevage, F-31326 Castanet-Tolosan, France; 2 Université de Toulouse, École Nationale Vétérinaire de Toulouse (ENVT), INRA, Interactions Hôtes - Agents Pathogènes (IHAP), F-31076 Toulouse, France; 3 INRA, Unité expérimentale 0321 Domaine de La Fage, F-12250 Roquefort sur Soulzon, France; 4 INRA, UR875 Castanet-Tolosan, France

## Abstract

Negative Energy Balance (NEB) is considered to increase susceptibility to mastitis. The objective of this study was to improve our understanding of the underlying mechanisms by comparing transcriptomic profiles following NEB and a concomitant mammary inflammation. Accordingly, we performed RNA-seq analysis of blood cells in energy-restricted ewes and control-diet ewes at four different time points before and after intra mammary challenge with phlogogenic ligands. Blood leucocytes responded to NEB by shutting down lipid-generating processes, including cholesterol and fatty acid synthesis, probably under transcriptional control of *SREBF 1*. Furthermore, fatty acid oxidation was activated and glucose oxidation and transport inhibited in response to energy restriction. Among the differentially expressed genes (DEGs) in response to energy restriction, 64 genes were also differential in response to the inflammatory challenge. Opposite response included the activation of cholesterol and fatty acid synthesis during the inflammatory challenge. Moreover, activation of glucose oxidation and transport coupled with the increase of plasma glucose concentration in response to the inflammatory stimuli suggested a preferential utilization of glucose as the energy source during this stress. Leucocyte metabolism therefore undergoes strong metabolic changes during an inflammatory challenge, which could be in competition with those induced by energy restriction.

## Introduction

The transition from late gestation to early lactation is the most metabolically challenging physiological stage in dairy ruminants. Energy balance (EB) is an important parameter in dairy ruminants, defined as the difference between energy intake from feed and energy required for body maintenance, gestation and milk production. During the periparturient period, ruminants experience some degree of negative energy balance (NEB) due to the increased energy demand required to support fetal growth and lactation coupled with a reduced appetite and dry matter intake^1^. Indeed, NEB leads to extensive mobilization of lipids from adipose tissue and results in increased concentrations of circulating non-esterified fatty acids (NEFA) and ketone bodies such as β-hydroxybutyrate (BHB)^2^.

Increased susceptibility to mastitis during the peripartum period has been widely documented, as reviewed by Pyörälä^3^. Large-scale studies have also demonstrated an association between blood NEFA and BHB and the risk of mastitis during early lactation^4, 5^. In addition, several *in vitro* studies have reported an impairment of immune functions in response to increased concentrations of NEFA or BHB in the culture milieu. For instance, Scalia *et al*.^6^ showed that high concentrations of NEFA reduced bovine polymorphonuclear cell (PMN) viability *in vitro*.

Little is known about the effect of NEB on blood gene expression and the response to an intramammary challenge. Moyes *et al*.^7^ compared the expression of 20 genes measured by qPCR in blood PMN between NEB and control-diet cows (n = 5) during a *Streptococcus uberis* intramammary challenge. The small number of differentially expressed genes did not allow the identification of differential pathways and an understanding of underlying biological mechanisms between blood immune response and NEB but instead opened the way to further studies on the biological basis for this association.

Transcriptome sequencing (RNA-seq) technologies provide a unique opportunity to analyze changes in gene expression across the entire expressed genome without a priori knowledge^8^. This technology has distinct advantages over microarrays, including the sensitive detection of all expressed genes without the need to generate an array of probes based on a known sequence, virtually no background noise, and a much higher dynamic range. RNA-seq has recently been widely used in domestic animals in order to identify the differentially expressed genes (DEGs) and novel transcript units.

A very limited number of studies related to NEB or mastitis traits have examined these questions using RNA-seq technology. Jin *et al*.^9^ showed differences in miRNA expression in bovine mammary epithelial cells challenged with *E. coli* or *S. aureus*. McCabe *et al*.^10^ studied the effect of NEB in bovine liver by using RNA-seq. However, to our knowledge, the effect of a NEB or a mammary inflammatory challenge has never been investigated on blood cells using RNA-seq technology. The objective of this study was therefore to describe and to compare the effects of a NEB and those of a concomitant inflammatory challenge on blood cell transcriptomes using RNAseq. For that purpose, we performed RNA-seq analysis of blood cells in energy-restricted ewes and control-diet ewes at four different sampling time points during an inflammatory challenge. The results described herein provide a significant advance in our knowledge of metabolic changes in blood cells during energy restriction and an inflammatory challenge of the mammary gland.

## Results

A total of 4,888 million paired-end reads were obtained from the transcriptome sequencing of the 96 blood samples analyzed (Subdata 1). Neither diet nor sampling time had any effect on the number of reads. The dataset obtained with FeaturesCount showed sample counts for 25,197 genes. After descriptive analysis, one outlier sample was removed from the dataset.

### Effect of diet and inflammatory challenge on phenotypic indicators

Results of the phenotype analysis are shown in Subdata 2. Ewes in the first month of lactation that were subjected to energy restriction had decreased body weight (BW), body condition score (BCS), milk yield and an increased fat-to-protein (F:P) ratio when compared to PEB ewes. Blood analyses revealed that NEB ewes also had higher BHB and NEFA concentrations and lower glucose concentrations than PEB ewes. However, no difference was observed between groups for insulin or T3 concentrations. Energy restriction had no significant effect on milk SCS, and total or subtype blood leucocyte concentrations.

The inflammatory mammary challenge induced a slight increase in glucose concentration. Statistically, a decrease in BW was also observed upon the inflammatory challenge; however, this difference was mainly observed in NEB ewes. Moreover, BW measurements were taken at an interval of 4 days around the inflammatory challenge, and the decrease in BW around this period is most probably due to energy restriction. The inflammatory challenge had no effect on BCS, F:P ratio or milk yield. No difference was observed either in BHB, NEFA, insulin, or T3 concentrations in the early response to the inflammatory challenge. As expected, the challenge elicited a strong increase in milk SCS and a decrease in total blood leucocytes. The number of all types of blood leucocytes was decreased in response to the inflammatory challenge (Subdata 2). However, neither the diet nor the inflammatory challenge had an effect on the blood cell composition. Blood cell percentages according to the diet and the inflammatory challenge are presented in Subdata 3.

### Differential expression of genes between PEB and NEB ewes

**Figure 1:**
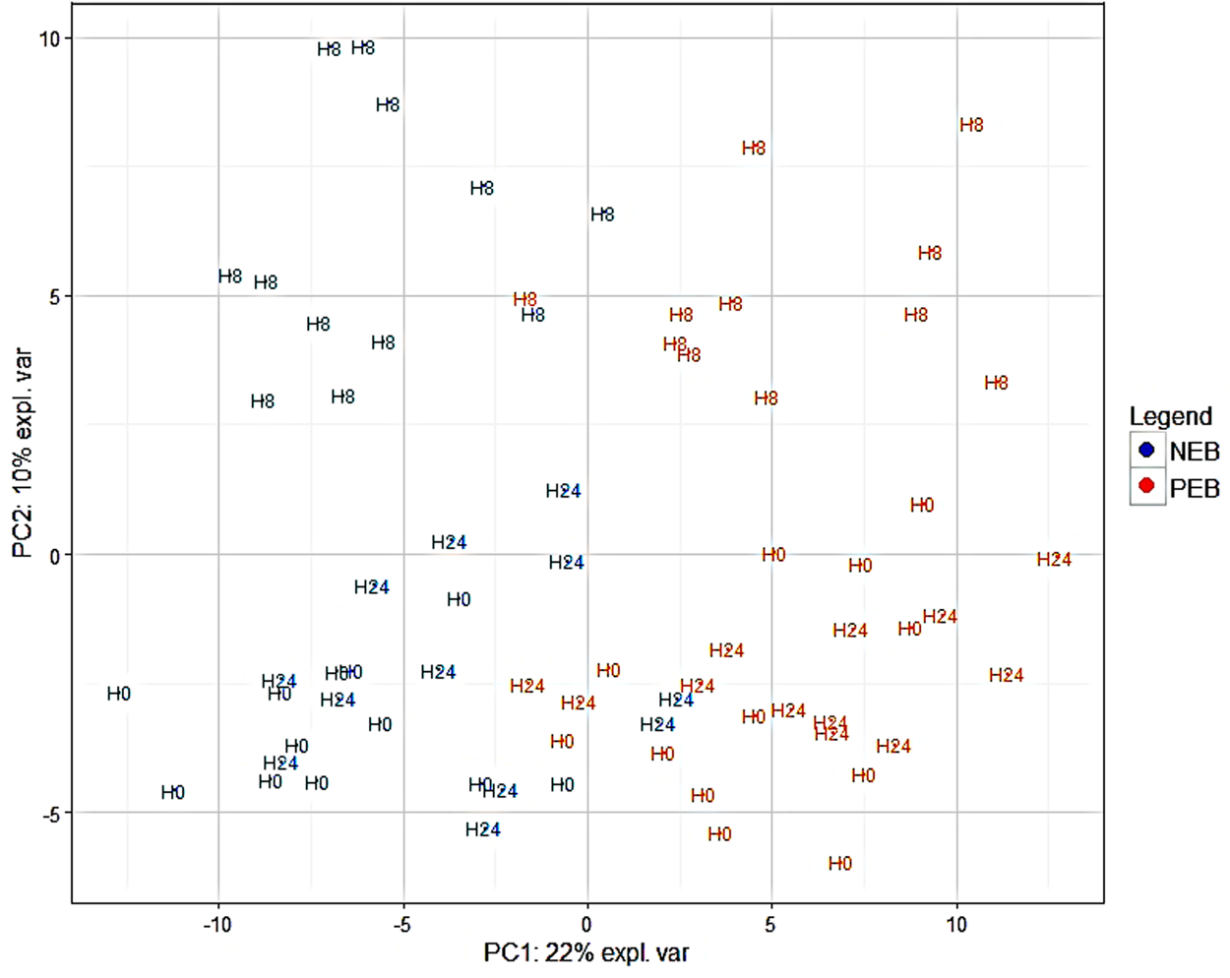
PCA performed on the 191 DEGs in response to energy restriction. The orange and blue colors indicate samples from Positive Energy Balance (PEB) and Negative Energy Balance (NEB), respectively. H0, H8 and H24 indicate the sampling hour relative to the inflammatory challenge.

We used three time points to obtain a strong list of DEGs in response to energy restriction (H0, H8, H24). The effect of the inflammatory challenge was corrected by the statistical model. A total of 191 genes were found to be differentially expressed in response to energy restriction (NEB vs. PEB at three sampling time points). Of these, 84 genes were up-regulated and 107 genes were down-regulated by NEB. Using Ingenuity Pathway Analysis®, a total of 183 genes were mapped or recognized based on annotation to human or mouse orthologs within the IPA Knowledge Base. PCA performed on the 191 DEGs in response to energy restriction are presented in Fig. 1. The first component on the PCA explained 22% of the variability between the samples and clearly discriminated between the two diets, supporting the hypothesis that the 191 genes were differential between the two diets.

Top canonical and signaling pathways (FDR < 0.05, ratio > 0.1) are reported in Table 1. At the pathway level, the s*uperpathway of cholesterol biosynthesis* was the most significant pathway modified in response to energy restriction. Eight DEGs associated with this pathway were all down-regulated: *CYP51A1* (FC = 0.8), *DHCR24* (FC = 0.9), *FDFT1* (FC = 0.86), *HMGCS1* (FC = 0.83), *IDI1* (FC = 0.92), *LSS* (FC = 0.83), *MVD* (FC = 0.86) and *SQLE* (FC = 0.82). A subset of those same DEGs supported the down-regulation of the *Cholesterol biosynthesis* and the *Mevalonate Pathway*. On the other hand, *TNFR2 signaling* was also inhibited. Three DEGs were associated with this pathway: *MAPK8* (FC = 0.82), *NFKB2* (FC = 0.92) and *BIRC3* (FC = 0.91).

**Table 1:** Top canonical and signaling pathways among Differentially Expressed Genes (DEGs) in response to energy restriction (NEB vs. PEB) with a q-value < 0.05 and a ratio > 0.1.

Ingenuity Canonical Pathways	q-value^1^	Ratio^2^	Up/Down regulated	DEG
*Superpathway of Cholesterol Biosynthesis*	7.6E-05	0.10	0/8	MVD, SQLE, FDFT1, DHCR24, IDI1, LSS, HMGCS1, CYP51A1
*Cholesterol Biosynthesis*	9.1E-04	0.13	0/5	SQLE, FDFT1, DHCR24, LSS, CYP51A1
*Mevalonate Pathway I*	4.6E-02	0.11	0/3	MVD, IDI1, HMGCS1
*TNFR2 Signaling*	5.0E-02	0.10	0/3	MAPK8, NFKB2, BIRC3

^1^The q-value shows the significance of the enrichment of a function within the DEGs, adjusted by Benjamini and Hochberg’s FDR.

^2^Ratio of DEGs/number of genes in the pathway.

We next looked for the upstream transcriptional regulators that could explain the observed gene expression changes and elucidate the biological activities due to NEB. The most significant upstream regulators, including peroxisome proliferator-activated receptor alpha (*PPARα*) and sterol regulatory element-binding transcription factor (*SREBF*) 1 and 2, are shown in Subdata 4. *PPARα*, which is a major regulator of lipid metabolism in the liver, was the most significant upstream regulator and was predicted to be activated (p-value of overlap = 3.21E-13, activation z-score = 2.56). In our dataset, *PPARα* expression tended to be down-regulated (FC = 0.8) with an adjusted p-value close to significance (p-value = 0.004, q-value = 0.1). PPARα was predicted in interaction with 23 DEGs, including *CPT1A* and *PDK4*, which were among the most up-regulated DEGs in the dataset.

SREBF 1 and 2 functions were predicted as being down-regulated, according to their association with 11 and 8 DEGs (p-value of overlap = 2.27E-7, activation z-score = −2.16 and p-value of overlap = 3.65E-8, activation z-score = −2.62, respectively). In our dataset, *SREBF 1* and *2* expressions were down-regulated (both FC = 0.92) with a p-value of 0.03 (q-value = 0.3) and 0.006 (q-value = 0.1), respectively. These transcription factors are major regulators of cholesterol synthesis and most of their predicted target molecules were DEGs described above in the inhibition of the *superpathway of cholesterol biosynthesis* (*CYP51A1*, *FDFT1*, *HMGCS1*, *IDI1*, *LSS*, *MVD* and *SQLE*). *FADS1*, which encodes for fatty acid desaturase, was down-regulated in NEB ewes and predicted to be down-regulated by SREBF1.

### Differential expression of genes in response to the inflammatory challenge

After 10 days, ewes were challenged by an intramammary injection of a combination of MAMPS (Pam_3_CSK_4_ and MDP) to induce a mammary inflammation. When comparing the gene expression profiles at 0 and 8 h after the challenge, a total of 3482 genes were differentially expressed and of these, 1641 genes were down-regulated, whereas 1841 genes were up-regulated. A total of 3101 genes were recognized by IPA for functional analysis.

Top canonical and signaling pathways (q-value < 0.01, ratio > 0.1) are reported in Table 2. Pathways playing major roles in the immune response like *B Cell Receptor Signaling, IL-6 Signaling, IL-10 Signaling, TNFR1 Signaling, Toll-like Receptor Signaling, Leukocyte Extravasation Signaling, iNOS Signaling* were highly significantly activated in response to the inflammatory challenge. *DNA Methylation and Transcriptional Repression Signaling* was also highly activated by the inflammatory challenge. On the other hand, pathways related to reparation of DNA like *ATM Signaling, DNA Double-Strand Break Repair by Homologous Recombination* and *Role of BRCA1 in DNA Damage Response* were inhibited.

**Table 2:** Top canonical and signaling pathways among Differentially Expressed Genes (DEGs) in early response (H + 8) to the inflammatory challenge with a q-value < 0.01 and a ratio > 0.1.

Ingenuity Canonical Pathways	q-value^1^	Ratio^2^	Up/Down regulated
*B Cell Receptor Signaling*	2.45E-04	0.27	38/11
*DNA Methylation and Transcriptional Repression Signaling*	3.72E-04	0.60	11/1
*ATM Signaling*	2.24E-03	0.36	9/12
*3-phosphoinositide Degradation*	2.24E-03	0.26	27/13
*Role of BRCA1 in DNA Damage Response*	5.37E-03	0.31	9/15
*D-myo-inositol-5-phosphate Metabolism*	5.89E-03	0.25	25/13
*IL-6 Signaling*	5.89E-03	0.27	25/6
*IL-10 Signaling*	5.89E-03	0.31	17/5
*TNFR1 Signaling*	5.89E-03	0.35	10/7
*DNA Double-Strand Break Repair by Homologous Recombination*	5.89E-03	0.57	1/7
*Cyclins and Cell Cycle Regulation*	5.89E-03	0.30	16/7
*D-myo-inositol -Tetrakisphosphate Biosynthesis*	5.89E-03	0.25	21/13
*Toll-like Receptor Signaling*	6.31E-03	0.30	14/8
*Leukocyte Extravasation Signaling*	6.92E-03	0.23	32/14
*Superpathway of Inositol Phosphate Compounds*	6.92E-03	0.22	32/16
*iNOS Signaling*	7.24E-03	0.34	13/3
*Estrogen Receptor Signaling*	7.59E-03	0.25	18/14

^1^The q-value shows the significance of the enrichment of a function within the DEGs, adjusted by Benjamini and Hochberg’s FDR.

^2^Ratio of DEGs/number of genes in the pathway.

### Overlapping features between response to energy restriction and the early response to the inflammatory challenge

**Figure 2:**
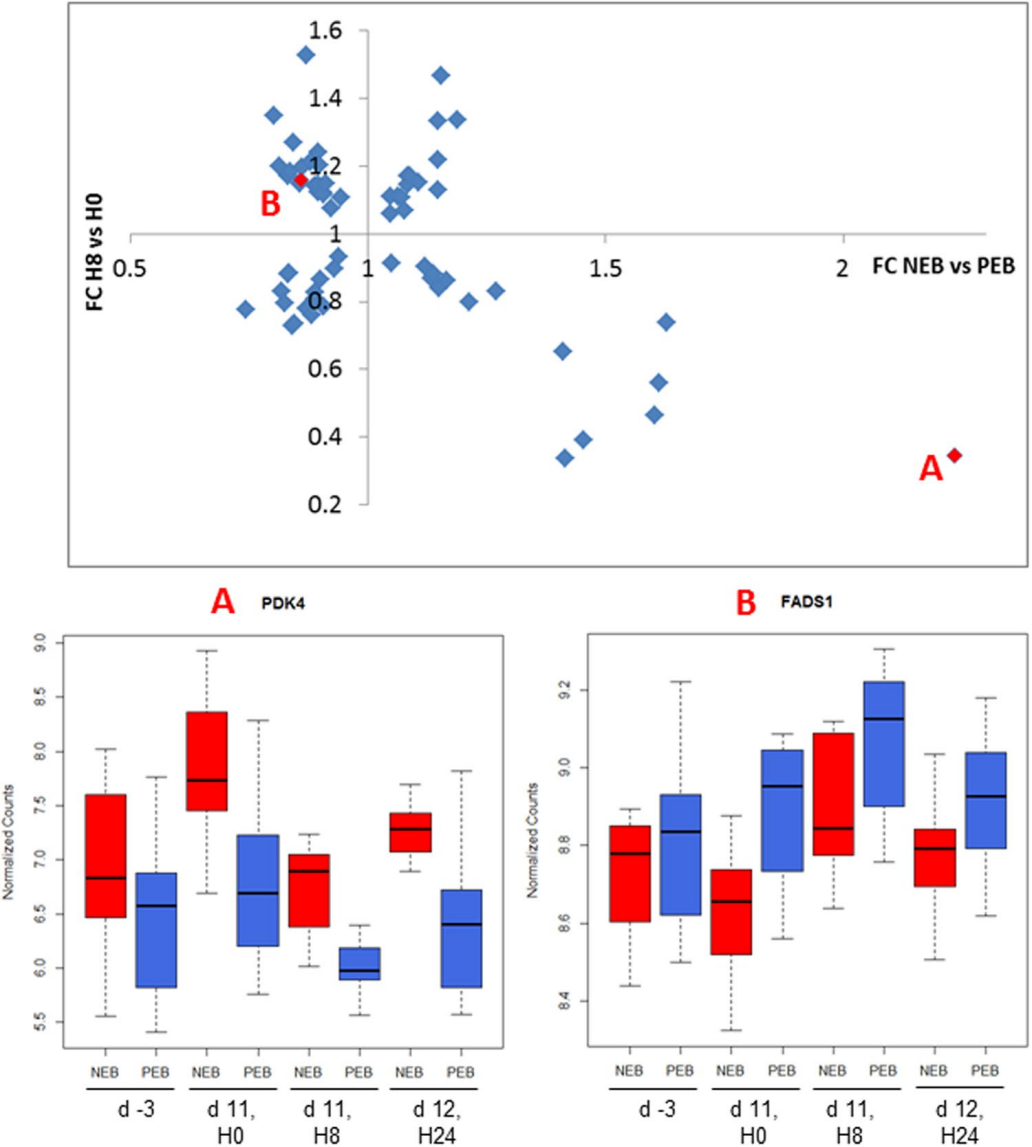
Fold change of the DEGs (q-value < 0.05) in response to energy restriction according to the fold change of the DEG (q-value < 0.05) in early response to inflammatory challenge. Boxplots show normalized counts of *FADS1* and *PDK4* genes in blood cells of Negative Energy Balance (NEB) ewes (red, n = 12) and Positive Energy Balance (PEB) ewes (blue, n = 12) at four different time points. Day time points are related to the first day of energy restriction (d 0) and hour time points are related to the inflammatory challenge (H0).

There was no significant gene in the Diet X Challenge interaction, suggesting that there was no straightforward modification of the response to inflammation according to diet, given the power of the current design. However, when comparing the two lists of DEGs, we identified 64 genes in common between the response to energy restriction and the early response to the inflammatory challenge. The list of 64 genes was further scrutinized to study overlapping (or conflicting) biological features between the two types of challenges, i.e., low energy diet vs. MAMPs-induced inflammation. The list was analyzed upon uploading in IPA. Figure 2 shows the relationship between changes due to energy restriction and those due to the early response to the inflammatory mammary challenge of the mammary gland. Several DEGs implicated in metabolic processes were present. Among them, *PDK4*, which was one of the DEGs with the highest upregulation in response to energy restriction, was down-regulated in early response to the inflammatory challenge. Fatty acid desaturase (*FADS1*) and Mevalonate Diphosphate Decarboxylase (*MVD*) were down-regulated in NEB ewes and up-regulated in response to the inflammatory challenge.

**Figure 3:**
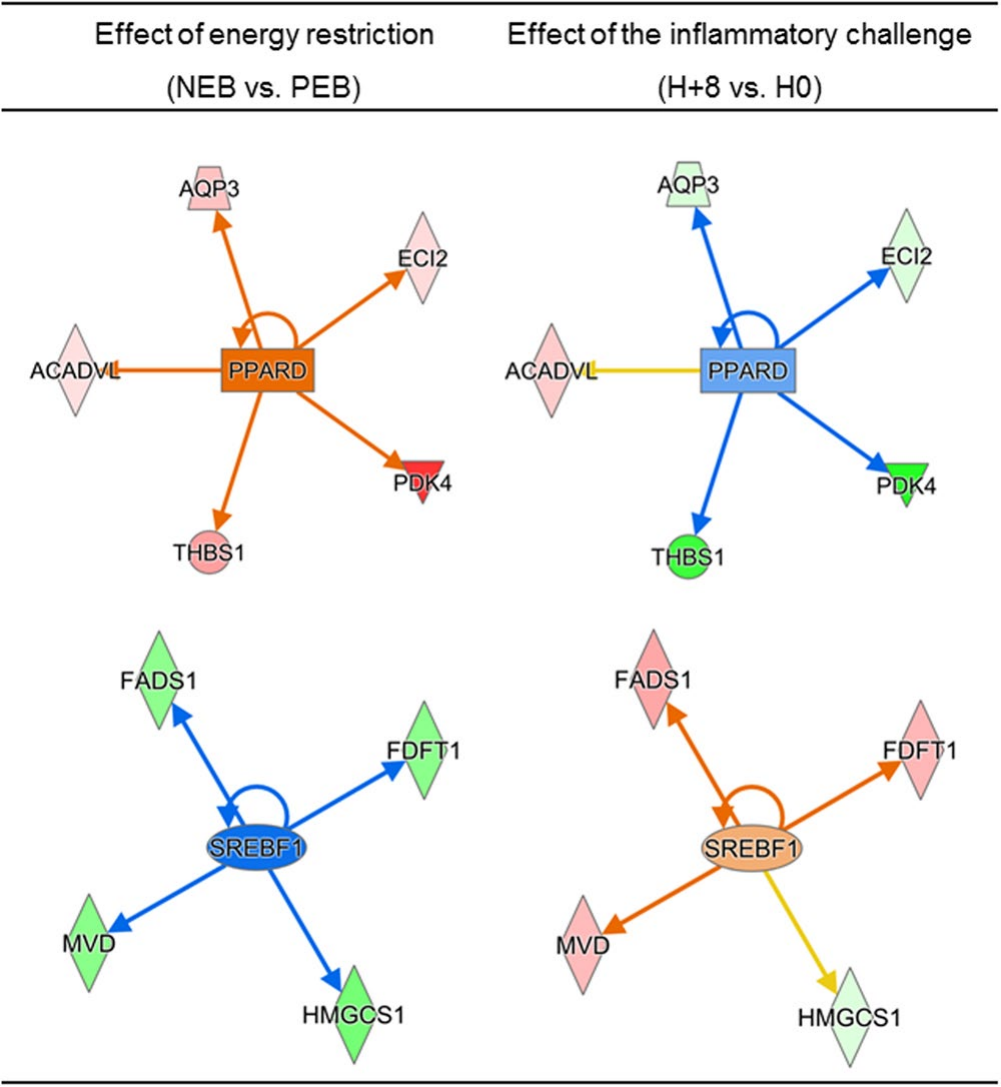
Predicted transcription regulators related to DEGs (q-value < 0.05) for both response to energy restriction and early response to the inflammatory challenge. Molecules highlighted in green were down-regulated and molecules highlighted in red were up-regulated.

Within the list of 64 DEGs, *PPARD* and *SREBF1* were predicted to be the most differential transcription regulators in response to energy restriction and early response to inflammatory challenge (Fig. 3). On the one hand, it was predicted that *PPARD* expression would be activated in response to energy restriction (z-score = 2.4) and inhibited in early response to the inflammatory challenge (z-score = −0.8). The predicted inhibition in response to the challenge was not consistent with a change in gene expression; indeed, *PPARD* was part of the list of 64 DEGs common to the responses to energy restriction (q-value = 0.03, FC = 1.09) and inflammatory challenge (q-value = 4.65E-08, FC = 1.17).

On the other hand, it was predicted that *SREBF 1* would be inhibited in response to energy restriction (z-score = −2.2) and activated in early response to the inflammatory challenge (z-score = 1.0). Those predictions were in agreement with observed FC. Indeed, as described previously, *SREBF1* had a tendency to be down-regulated in response to energy restriction in our dataset. *SREBF1* also had a tendency to be up-regulated in response to inflammatory challenge (q-value = 0.07, FC = 1.12). It was predicted that *SREBF1* was related to *FADS1*, *FDFT1*, *HMGCS1* and *MVD*, and these molecules were down-regulated in response to energy restriction and up-regulated in the early response to the inflammatory challenge, except *HMGCS1*, which was slightly up-regulated in response to the inflammatory challenge. *FDFT1*, *HMGCS1* and *MVD* are DEGs that are related to cholesterol biosynthesis described above.

### RT- qPCR validation of the differentially-expressed genes in response to energy restriction and the inflammatory challenge

RT- qPCR was used to confirm the response of nine metabolic genes to energy restriction and to the inflammatory challenge on a new set of samples collected during the same experiment (Table 3). Five genes involved in the cholesterol synthesis were tested: *HMGCS1*, *MVD*, *FDFT1*, *CYP51 A1* and *SQLE*. Their down-regulation in response to the energy restriction was confirmed by qPCR, whereas upregulation was shown in response to the inflammatory challenge except for *CYP51 A1*. *CPT1A*, *PDK4*, *FADS1* and *BDH1* were amongst the most differential genes in response to energy restriction and their expression changes were confirmed in response to both challenges, except CPT1A downregulation during the inflammatory challenge.

**Table 3:** Analysis of variance (linear mixed model) of the effect of diet (Positive Energy Balance: PEB vs Negative Energy Balance: NEB) and of the inflammatory challenge (after vs before) on RT-qPCR data measured in a different set of samples. Variation between two conditions (B compared to A) was calculated as a relative difference in lsmeans: (lsmeansB-lsmeansA)/lsmeansA. NEB ewes (n = 12) were compared to PEB ewes (n = 12). Gene expression after the inflammatory challenge (n = 24) was compared to gene expression before the inflammatory challenge (n = 24). Significant effects were highlighted by stars: ^∙^P < 0.1, *P < 0.05, **P < 0.01 and ***P < 0.001.

Trait	Effect of diet			Effect of inflammatory challenge		
	lsmeans	lsmeans	Relative effect	lsmeans	lsmeans	Relative effect
	PEB	NEB	(NEB vs PEB)	Before	After	(After vs Before)
BDH1	1.07	0.78	−27.0*	0.81	1.09	+34.8^∙^
CPT1A	1.16	2.01	+72.7***			
CYP51A	1.11	0.87	−21.2*			
FADS1	1.41	1.12	−21.0.	0.95	1.54	+63.0**
FDFT1	1.42	1.11	−21.7*	0.98	1.65	+68.5***
HMGCS1	1.27	0.96	−24.8**	0.98	1.20	+22.7^∙^
MVD	1.25	0.97	−22.5*	0.88	1.37	+55.0**
PDK4	1.11	2.46	+121.9***	2.09	1.16	−44.5**
SQLE	1.36	1.01	−25.8**	1.00	1.40	+40.6**

## Discussion

One of the focuses of our study was to test the hypothesis that metabolic and signaling pathways are altered in ruminant blood cells during energy shortage and associated ketosis using periparturient ewes as a model. Biological parameters of NEB ewes showed decreased serum glucose concentration (−6%) and dramatically increased serum NEFA (+43%) and BHB (+97%) concentrations compared to PEB ewes. Results were consistent with previous data on a larger number of animals (n = 48) from the same design (e.g. ref. 11). These results suggest that our feed restriction model was appropriate for mimicking adaptations generally observed in postpartum high-yielding dairy species.

In the present experiment, the most differential pathway in response to energy restriction was the decrease observed in cholesterol synthesis (down-regulation of *CYP51 A1*, *DHCR24*, *FDFT1*, *HMGCS1*, *IDI1*, *LSS*, *MVD* and *SQLE*). The reaction catalyzed by HMGCS1 is the rate-determining step on the pathway for synthesis of cholesterol^12^, and HMGCS1 gene expression was confirmed downregulated in response to energy restriction by the qPCR validation. This decrease in cholesterol biosynthesis is also supported by a microarray study describing the down-regulation of cholesterol synthesis in the liver of feed-restricted lactating cows compared with cows fed ad libitum^13^, suggesting that blood leucocytes and hepatocytes share similar alterations of cholesterol metabolism due to energy restriction. To our knowledge, there is no previous study showing such a decrease in cholesterol synthesis in blood cells in response to energy restriction. Similarly to hepatocytes^14^, blood cells therefore achieve cholesterol hemostasis through the control of the expression of key cytosolic enzymes required for their endogenous biosynthesis.

Moreover, expression of genes involved in synthesis of fatty acids and triglycerides was also affected by energy restriction. Indeed, the synthesis of fatty acids from acetyl-CoA and malonyl-CoA is carried out by fatty acid synthase, FAS. Fatty acid synthase is encoded by the *FASN* gene, which had a tendency to be down-regulated (q-value = 0.1, FC = 0.92) in our dataset and in accordance with Loor *et al*.^13^. As previously described by Faulconnier *et al*.^15^ in the adipose tissue of lactating goats, fatty acid desaturation was also affected by energy restriction. Indeed, *FADS1* and *SCD*, two genes encoding fatty acid desaturases, were both affected by energy restriction. *FADS1* was significantly down-regulated in both sets of samples, and *SCD* had a tendency to be down-regulated (q-value = 1.7E-03, FC = 0.86 and q-value = 0.06, FC = 0.78, respectively).

According to our IPA analysis, decreased activities of lipogenic enzymes in blood cells during the early lactation NEB were most probably under the control of sterol regulatory element-binding protein *SREBF* 1 and 2 transcription factors. Such a role of *SREBF* 1 and 2 transcription factors has already been shown in the liver tissue of energy-restricted cattle^13^.

On the other hand, expression of genes involved in NEFA oxidation tends to be activated in response to energy restriction. Indeed, *CPT1A*, which encodes a key regulatory enzyme in fatty acid oxidation, was one of the most up-regulated DEGs in response to energy restriction. This up-regulation was significant in both sets of samples, in contrast to Loor *et al*.^13^ who did not find any change in *CPT1A* expression in the liver of ketotic cows. CPT1 is anchored in the outer membrane of mitochondria and catalyzes the formation of long-chain acyl-carnitine, which is then allowed to pass through the inner mitochondrial membrane and is thus committed to β-oxidation in the mitochondria. Moreover, *ACADVL* and *ECI2*, which code for enzymes specific to the beta-oxidation of long-chain fatty acids and unsaturated fatty acids, respectively, were both up-regulated. Our study therefore showed that genes involved in fatty acid β-oxidation were up-regulated in NEB ewes to provide acetyl-CoA subsequently used in the TCA cycle. Consequently, fatty acids were possibly used as the main fuel source in blood cells during fasting, as previously shown in human blood leucocytes^16^.

Ketogenesis is a mitochondrial process by which acetyl-CoA, mostly derived from the oxidation of fatty acids, is converted through four reactions into acetoacetate, BHB and acetone, all of which are commonly called ketone bodies. The rate-limiting step of ketone body synthesis is the condensation of acetyl-CoA and acetoacetyl-CoA into HMG-CoA by mitochondrial 3-hydroxy-3-methylglutaryl-CoA synthase 2 (HMGCS2)^17^. Since ketogenesis is mainly hepatic, *HMGCS2* is one of the most highly induced genes in liver during fasting^17^. In our study, we did not observe any change in *HMGCS2* expression in blood cells. Acetoacetate is then liberated by HMG-CoA lyase (HMGCL) from HMG-CoA. Most acetoacetate is further metabolized by the liver into BHB by β-hydroxybutyrate dehydrogenase (BDH1). Ketone bodies are then used by different tissues as fuels, thus saving glucose. In particular, BHB is converted back into acetoacetate by BDH1 once it is taken up by a target tissue. BDH1 was surprisingly down-regulated during energy restriction in our study and this down-regulation was confirmed by the qPCR data in a new set of samples. No difference in other enzymes taking part in BHB oxidation expression was observed, suggesting that ketone bodies were not the main source of energy in blood cells during energy restriction in our study.

Bouwens *et al*. studied the effects of fasting on human peripheral blood mononuclear cell gene expression profiles^16^. In accordance with our study, they found that the gene encoding pyruvate dehydrogenase kinase isoform 4 (PDK4) showed the biggest change during fasting. PDK4 inactivates pyruvate dehydrogenase (PDH), inhibiting utilization of pyruvate for acetyl-CoA synthesis, hence blocking glucose oxidation and favoring fatty acid oxidation to generate energy^18^. This gene is known to be regulated by PPARα. Activation of PPARα especially occurs when NEFA concentrations are increased in situations such as fasting and is essential for metabolic adaptation by up-regulating genes for beta-oxidation and ketogenesis, and by down-regulating energy expenditure. Bouwens *et al*. showed that the expression of all PPARα target DEGs was up-regulated during starvation, similar to results of our study^15^. However, no difference in *PPARα* expression could be observed due to fasting in their study. Similarly, *PPARα* expression was predicted to be activated by IPA in our study although it had a tendency to be down-regulated (q-value = 0.1, FC = 0.8).

Glucose uptake is controlled in part by the cell-surface expression of a family of glucose transporters (GLUT). The ubiquitous GLUT1 is the predominant glucose transporter in the bovine mammary gland and is encoded by the gene *SLC2A10*. This gene had a tendency to be down-regulated in our study (q-value = 0.07, FC = 0.8), in accordance with Tsiplajou *et al*.^19^ who showed a significant reduction on mRNA of GLUT1 in ovine mammary tissue in response to feed restriction. This observation once again supports a decrease of glucose utilization in blood leucocytes during energy restriction.

To conclude, this analysis suggested that blood leucocytes respond to NEB and ketosis by shutting down lipid-generating processes, including mevalonate, cholesterol and fatty acids synthesis, to a similar extent to what has been previously described for liver tissue. SREBF 1 and 2 seem to play a key role in the regulation of these metabolic processes. Furthermore, activation of several enzymes like PDK4 and CPT1 seemed necessary to block glucose oxidation and induce fatty acid or ketone body oxidation to generate energy, similarly to observations in human blood leucocytes.

No significant interaction between energy balance and the response to the inflammatory challenge on blood transcriptome was identified in our study. This could be explained by the lack of power in our study (2 diets * 2 time points * 12 ewes). However, 64 DEGs in response to energy restriction were also significantly differential in early response to the inflammatory challenge.

**Figure 4:**
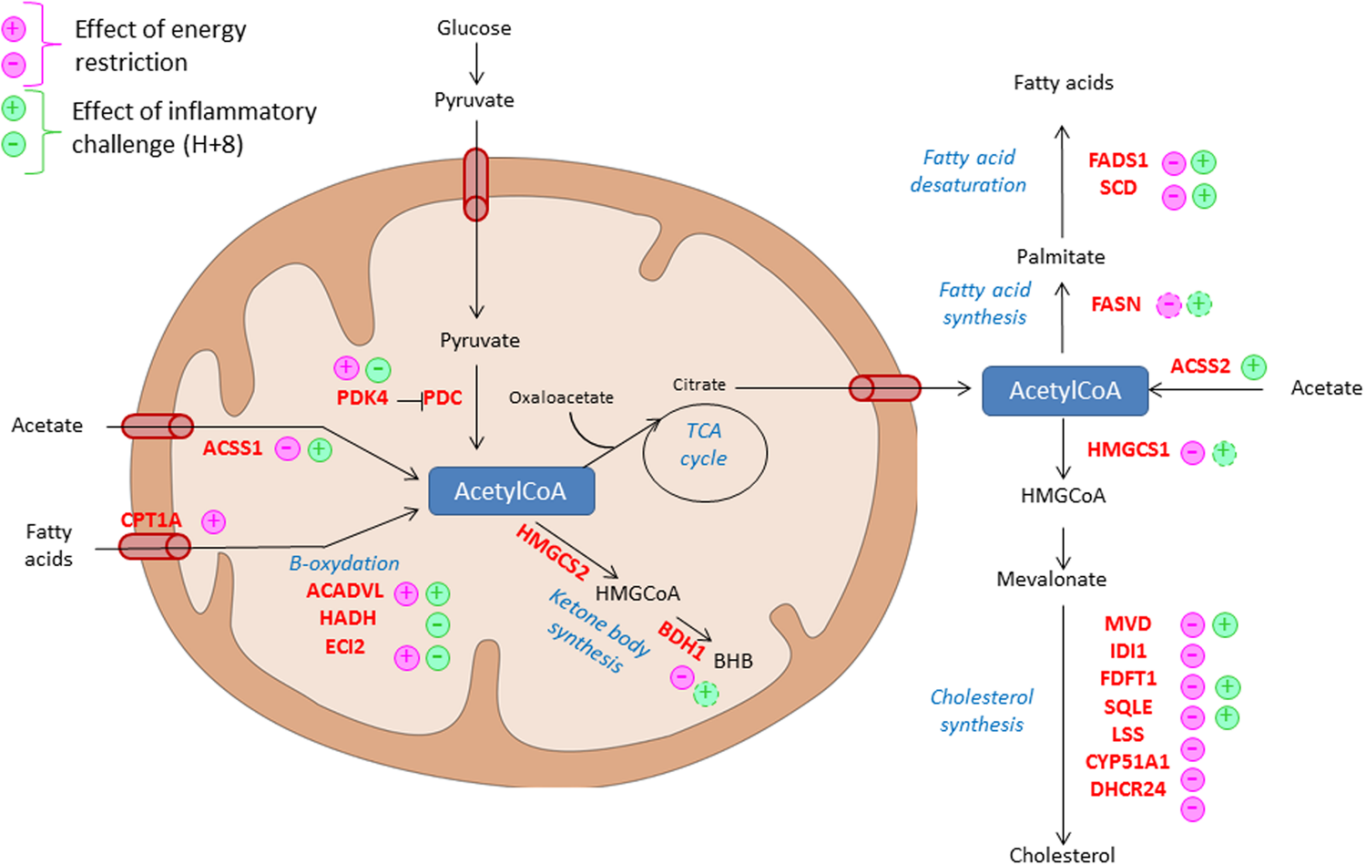
Summary diagram showing the effect of energy restriction and an inflammatory challenge on mitochondrial metabolic processes and their conflicting features. Pyruvate dehydrogenase kinase PDK4 inhibits the pyruvate dehydrogenase complex (PDC). Plus indicates up-regulated DEG and minus down-regulated DEG. Dotted plus or minus indicate DEG that had a tendency (q-value < 0.1) to be respectively up or down-regulated.

From this common list, several metabolic pathways were analyzed as being differential between the response to energy restriction and the response to the inflammatory challenge as shown in Fig. 4. In particular, *FDFT1* and *MVD* were confirmed up-regulated in response to the inflammatory challenge suggesting that cholesterol synthesis was activated. Moreover, genes involved in synthesis of fatty acids and triglycerides were also affected by the inflammatory challenge. Indeed, fatty acid synthase encoded by the *FASN* gene had a tendency to be up-regulated (q-value = 0.1, FC = 1.07). *SCD* and *FADS1*, which encode fatty acid desaturases, were both up-regulated in response to inflammatory challenge, despite small variations. The up-regulation of these lipid-biosynthetic pathways was in accordance with early isotopomer-enrichment studies that demonstrated that the activation of lymphocytes results in a rapid increase in the renewed biosynthesis of cholesterol and fatty acids^20^. According to this study, the synthesis of cholesterol could be an essential prerequisite for successful initiation and completion of the cell cycle in lymphocytes.

Furthermore, IPA predicted that *SREBF1* was a transcription factor differentially expressed in response to the two stresses, inhibited in response to energy restriction as described below and activated in response to the inflammatory challenge. These predictions were supported by trends in differential expression in our dataset (significant for diet and close to significance for challenge) and suggested that *SREBF1* could be the transcription factor responsible for activation of lipid-biosynthetic pathways in response to the inflammatory challenge. In accordance, Kidani *et al*.^21^ demonstrated that SREBPs were essential for CD8+ T cells to perform blastogenesis which resulted in attenuated clonal expansion during viral infection. These experiments all together suggest that metabolic reprogramming can be a key component during an inflammatory response due to its link with mitogenic signaling.

PPARγ was also one of the most differentially expressed transcription factors predicted by IPA in response to the two stresses. Moyes *et al*.^22^ did not detect any change in PPARγ expression in bovine mammary tissue challenged with *Streptococcus uberis* but suggested that its activity was probably decreased, as supported by the down-regulation of its known target genes. We did observe a down-regulation of PPARγ-known target genes. However *PPARγ* expression was up-regulated in response to the inflammatory challenge, which does not exclude a difference of activation of the protein explaining the PPARγ role in metabolic reprogramming during the inflammatory challenge.

Energy sources appeared also different in response to the inflammatory challenge from those observed in response to energy restriction. First, inflammatory challenge induces changes in genes expression involved in acetate catabolism. In ruminant animals, large amounts of acetate are synthetized by microorganisms in the rumen and need to be catabolized. Generally, mammalian acetyl-CoA synthesis from acetate is carried out by Acyl-CoA Synthetase Short-Chain Family Member (ACSS) 2 to support lipid synthesis in the cytosol and by ACSS1 to fuel ATP production in mitochondria^23^. *ACSS1* and *ACSS2* expression were both activated in response to the inflammatory challenge, whereas *ACCS1* expression was inhibited in response to energy restriction. Our hypothesis is that acetate production could have been decreased in response to the decrease in rumen activity during energy restriction. Similarly to glucose, a decrease in acetate concentration could be associated with its lesser use in blood cells. On the other hand, *ACSS1*, and especially *ACSS2* expression, could have been activated in response to the inflammatory challenge to support the need for acetyl-CoA and, consequently, lipid synthesis during inflammation.

Inflammatory challenge also induced expression changes in genes involved in fatty acid oxidation. Indeed, *ECI2, CPT1A* and *HADH*, which encode an enzyme belonging to the beta-oxidation pathway expressions were or had a tendency to be inhibited in response to the inflammatory challenge.

Finally, genes involved in glucose transport were also affected by the inflammatory challenge. Indeed, *SLC2A3* and *SLC2A10* encoding respectively GLUT3 and GLUT10 were both up-regulated in response to the inflammatory challenge (FC = 1.4 and FC = 1.8, respectively). However, no significant change was observed in GLUT1 transcription. These observations are in accordance with O’Boyle *et al*.^24^ who showed that endotoxin stimulation increased gene expression of GLUT3 and GLUT4 in bovine monocytes. On the other hand, *PDK4* expression was inhibited in response to inflammatory challenge as shown in both sets of data, in contrast with Hana Park and Nam Ho Jeoung *et al*.^25^ who showed that inflammation increases *PDK4* expression via the Jun N-Terminal Kinase (JNK). Jun is a transcription factor whose expression was down-regulated in response to the inflammatory challenge in our dataset. As described below, pyruvate dehydrogenase kinases play a critical role in the inhibition of the mitochondrial pyruvate dehydrogenase complex, especially when blood glucose levels are low and pyruvate can be spared for gluconeogenesis. Moreover, glucose concentration increased in response to the inflammatory challenge as shown in the phenotypic analysis. Other researchers have observed elevated plasma glucose concentrations after *E. coli* LPS or *Streptococcus uberis* infusion^26, 27^. Those researchers attributed the increased circulating glucose concentrations to enhanced glycogenolysis. Since glucose is the primary fuel used by bovine leukocytes^28^, glycogenolysis could be essential to allow immune activity. There is no doubt that further studies are needed to disentangle this hypothesis and to better understand the regulation of the pyruvate dehydrogenase complex in leucocytes during an inflammatory challenge.

## Conclusion

Our study indicated that the inflammatory challenge of the mammary gland induced a strong transcriptomic response in blood cells and mainly activated immune pathways. No interaction between energy balance and the response to the inflammatory challenge has been identified. However, energy restriction and inflammatory challenge induced some opposite effects in expression of genes involved in metabolic pathways. Indeed, this analysis suggests that blood leucocytes respond to negative energy balance and ketosis by shutting down lipid-generating processes, including mevalonate, cholesterol and fatty acid synthesis. On the other hand, lipid synthesis seemed increased in response to the inflammatory challenge. In both cases, the transcription factor SREBF 1 appeared to play a key role in the regulation of these metabolic processes, as predicted by IPA and suggested by our dataset. Pathway analysis also suggested an activation of fatty acid oxidation in response to energy restriction and its possible inhibition in response to the inflammatory challenge. Leucocyte metabolism therefore underwent strong changes during an inflammatory challenge, which could be in competition with those induced by energy restriction. Overall, this study suggested a difference in glucose utilization in response to the two stresses. Indeed, the strong increase in PDK4 transcription in response to energy restriction suggested an inhibition of glucose oxidation, whereas its decrease in response to inflammatory challenge, coupled with the increase of plasma glucose concentration and glucose transporter expression, suggested that glucose utilization and oxidation were enhanced by the inflammatory stress. Two hypotheses can be formulated in light of these results. Either glucose oxidation increased because glucose concentration was no longer low, or glucose concentration increased to meet the increasing need of glucose in leucocytes during the inflammatory challenge. Further studies are needed to disentangle these hypotheses and to better understand leucocyte metabolism during an inflammatory stress.

## Methods

All procedures involving animals received approval from the Ethics Committee on Animal Experimentation of Toulouse (France), with all applicable provisions established by the European directive 2010/63/UE. All methods were performed by approved staff members in accordance with the relevant standard operating procedures approved by the above mentioned ethics committee. All animals used in this study were handled in strict accordance with good clinical practices and all efforts were made to minimize suffering.

### Experimental design

**Figure 5:**
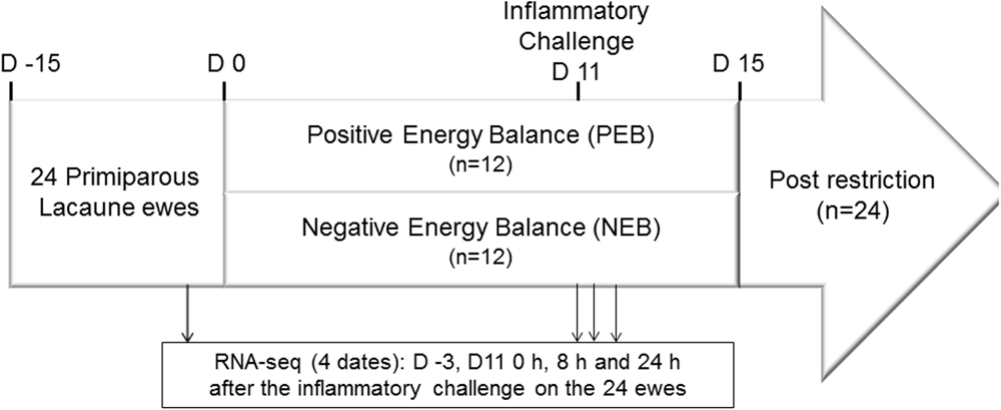
Experiment timeline.

A detailed description of the experimental design can be found in Bouvier-Muller *et al*.^11^. Briefly, 24 primiparous Lacaune ewes from the two genetic lines, High and Low-SCS, divergently selected for high and low mastitis resistance as described in Rupp *et al*.^29^ were used. Two weeks after lambing, the ewes were randomly assigned to either the Negative Energy Balance (NEB) or Positive Energy Balance (PEB) groups to obtain two groups of 12 ewes. The ewes assigned to the PEB treatment were fed a control diet composed of a standard total mixed ration for ad libitum intake. Ewes assigned to the NEB regimen were restricted to 60% of the calculated net energy requirements based on individual body weight and milk production. As shown in Fig. 5, the first day of restriction was referred to as d 0 in the experiment timeline. Ewes were maintained under their specific regimen for 15 days. After 11 days of feed restriction and following the morning milking, all ewes were injected with a solution of phlogogenic agents (Pam3CSK4 and MDP) into the healthiest half-udder. A detailed description of the phenotype collection can be found in Bouvier-Muller *et al*.^11^.

### Flow cytometry enumeration of leukocyte subsets in whole blood

Blood was collected in EDTA-coated tubes. Sixty microliters of whole blood were incubated with cocktails of pre-conjugated monoclonal antibodies for 20 minutes at room temperature in the dark. Red blood cell lysis was achieved by adding 500 µL of MACS RBC Lysis Solution (Miltenyi Biotec) for 10 minutes. Osmolarity of the solution was restored using FACS buffer (Dulbecco’s modified PBS, calcium and magnesium free, 2.5 mM EDTA, 0.1% BSA, pH 7.2) to a final volume of 1.2 mL, thus giving a 1:20 dilution of the original blood volume. Blood leucocytes were first gated based on CD45 expression (clone 1.11.32, Biorad, USA), and granulocytes and mononuclear cells were identified based on forward and side scatter distribution. A combination of CD2 (clone CC42, Biorad, USA) and CD14 (clone TÜK4, Biorad, USA) allowed identification of monocytes as CD2^neg^ CD14^pos^, T cells as CD2^pos^, CD14^neg^, and non T lymphocytes as CD2^neg^ CD14^neg^ cells (Subdata 6). Samples were acquired using a MACSQuant Analyzer capable of absolute cell count (Miltenyi Biotec). Total cell numbers per milliter in the original sample were calculated by multiplying the number of cells per mL in the CD45^pos^ gate by the dilution factor (1:20).

### Statistical analysis of phenotype and leukocyte enumeration

Analyses of variance using linear mixed models with the R (3.1.2) Bioconductor package nlme (3.1) were applied to determine the effects of the diet and the inflammatory challenge on the different phenotype and leukocyte enumeration. To test the effect of the energy restriction, the model included the fixed effects of Diet (DIET; PEB and NEB), genetic line (LINE; SCS+ and SCS−), and the random animal effect. To test the effect of the inflammatory challenge, we compared data before and after the challenge. The model included Diet (DIET; PEB and NEB), Genetic line (LINE; SCS+ and SCS−), Challenge (Challenge; Before or After) and the random animal effect. Statistical differences were declared as significant and highly significant at P < 0.05 and P < 0.01, respectively. Trends toward significance are discussed at P < 0.10. For an effect with a P < 0.10, lsmeans were extracted with the lsmeans package (2.20). Variation between two conditions (B compared to A) was then calculated as a relative difference in lsmeans: (lsmeansA-lsmeansB)/lsmeansA.

### RNA extraction

Blood samples were collected by jugular venipuncture on d -3, on d 11 at 0 and 8 hours post-challenge, and on d 12 at 24 hours post-challenge, as shown in Fig. 5. Samples were drawn from each ewe into blood collection tubes (6-ml BD Vacutainer®) containing EDTA for plasma and were immediately placed on ice. Total RNA was isolated from blood samples using the commercially available NucleoSpin® RNA Blood kit, according to the manufacturer’s protocol. Obtained RNA was quantified by measuring the absorbance at 260 nm using a NanoDrop ND-1000 (NanoDrop Technologies, Wilmington, DE, USA) and integrity was checked by Bioanalyzer (Agilent Technologies, Santa Clara, CA, USA). The RNA integrity value (RIN) of the samples ranged between 7.1 and 9.

### Library preparation for Illumina sequencing

cDNA libraries were prepared from high quality RNA using an Illumina TruSeq RNA sample prep kit, following the manufacturer’s instruction (Illumina, San Diego, CA, USA). Samples were tagged to allow subsequent identification, amplified by polymerase chain reaction (PCR) and quantified by quantitative PCR (Agilent QPCR Library Quantification Kit). Individual RNA-seq libraries were sequenced in triplicate at 100 bp/sequence paired-end reads using an Illumina HiSeq 2500 sequencer (Illumina, TruSeq PE Cluster Kit v3, cBot and TruSeq BS Kit v3) at the GenoToul genomic platform (Castanet-Tolosan, France). Library positions were randomized in four different sequencing lanes to avoid confusing flow cell/lane effects.

### Quality Control, Mapping and Quantification

FastQC (v0.10.0) was used to assess the quality of raw sequencing data. Input reads were then aligned to the ovine reference genome (ENSEMBL v74) using STAR (v2.4.0). For the mapping, the number of multiple alignments allowed for a read was 10 (if exceeded, the read was considered unmapped). Intron length was allowed between 10 and 25000 nucleotides. The alignments were sorted with Samtools (v0.1.19). To compare the expression levels of genes across samples, raw counts for the genes were obtained using the FeaturesCount software package and the ENSEMBL v74 annotation of the ovine genome. For counting, the RNA sequencing was indicated as reversely stranded and paired end (fragments were counted instead of reads, and paired distances were checked). Only primary alignments were counted, and chimeric fragments were not included for summarization.

### Differential Expression Analysis

The R (3.3.1) package, DESeq2 (1.12.4), was applied to identify statistically significant differentially-expressed genes (DEGs). DESeq normalizes the count data from high-throughput RNA sequencing across samples, based on the hypothesis that most genes are not DE. DESeq uses a negative binomial distribution to model biological and technical variance for the count data. It allows testing for differential expression between two experimental conditions.

To determine DEGs according to DIET, blood RNAseq data collected at the three time points at day 11 and 12 were used. Indeed the separate time by time analyses provide only a few DEG, therefore all relevant time points were included in a multifactor analysis to increase the power of the analysis. The model included diet (DIET; PEB and NEB), genetic line (LINE; SCS+ and SCS−) and time point relative to challenge (DATE; H0, H8 and H24). To determine DEGs according to the inflammatory challenge, RNAseq data collected at the time point before (H0) and the time point after challenge (H8) were used. The model included diet (DIET; PEB and NEB), genetic line (LINE; SCS+ and SCS−) and time point (DATE; H0 and H8). Finally, data from the time points H0 and H8 were used to determine the DEGs according to the interaction, diet: inflammatory challenge. The model included diet (DIET; PEB and NEB), genetic line (LINE; SCS+ and SCS−), time point (DATE; H0 and H8) and interaction (DATE:DIET). The p-values were adjusted using the Benjamini and Hochberg method^30^. A corrected q-value of 0.05 was set as the threshold for DEGs in all analyses.

### Biological interpretations of the differentially expressed genes

Ingenuity® Systems Pathway Analysis (IPA, Ingenuity Systems, Redwood City, CA, USA; http://www.ingenuity.com) was used to identify canonical pathways and functional processes of biological importance within the list of DEGs. Each interaction in the Ingenuity® Knowledge Base is supported by previously published information. The DEGs (with a q-value ≤ 0.05) with their associated annotation (when present) and the log2 Fold Change were uploaded into IPA. The significance of the canonical pathway was measured with the p-value and the ratio of DEG/number of genes in the pathway. A corrected q-value of 0.05 and a ratio of 0.1 were set as the threshold for differential expressed canonical pathways to minimize false positives. IPA additionally provides a z-score that infers the activation state of the canonical pathway. IPA also allows predicting whether some transcriptional factors that are known to regulate the listed focus genes are overrepresented in the data; an overlap p-value that measures whether there is a statistically significant overlap between the dataset genes and the known target genes was also used to describe the significance of the predicted transcriptional factor.

BLAST (http://blast.ncbi.nlm.nih.gov/Blast.cgi) was used to identify a few DEGs that only had an Ensembl annotation. Cover and identification of 60% and 80%, respectively, were set as the thresholds for the validation of the blast.

### Reverse transcription quantitative polymerase chain reaction (RT-qPCR)

Nine genes involved in metabolic process were assessed by RT-qPCR on blood samples from 24 ewes which followed the same experimental design than the 24 ewes in the discovery step using the RNA-seq technique. After RNA extraction, cDNA was generated using the Superscript III First Strand Synthesis System Kit (Invitrogen) following the manufacturer’s instructions. Primer pairs were designed using Primer3plus^31^ (Subdata 5) and their specificity was checked with BLAST. Primers were synthesized commercially by Eurogentec. qPCR reactions were performed using Biomark (Fuidigm) at the GenoToul genomic platform (Castanet-Tolosan, France). All assays were carried out in duplicate and Ct values were averaged for technical replicates. The stability of 5 housekeeping genes was evaluated in the 96 samples using GeNorm software. The three most stable genes (*YWHAZ*, *SDH* and *GAPDH*) were retained for normalization. Fold changes were calculated by the delta- delta Ct method^32^. Statistical analysis was performed as detailed in the statistical analysis of phenotype section.

## Electronic supplementary material

Subdata figures

Subdata 1
