# Supplementary material for: Whole blood transcriptome analysis reveals potential competition in metabolic pathways between negative energy balance and response to inflammatory challenge
Source: Sci Rep. 2017 May 24;7:2379. doi: 10.1038/s41598-017-02391-y (PMC5443788; doi:10.1038/s41598-017-02391-y)

**Whole blood transcriptome analysis reveals potential competition in metabolic pathways between negative energy balance and response to inflammatory challenge**

Juliette Bouvier-Muller, Charlotte Allain, Guillaume Tabouret, Francis Enjalbert, David Portes, Céline Noirot, Rachel Rupp & Gilles Foucras

**Subdata 2.** Analysis of variance (linear mixed model) of the effect of diet (PEB vs NEB) measured in NEB (n=12) and PEB (n=12) ewes, and of the inflammatory challenge measured before (n=24) and after challenge (n=24) on different phenotypic traits. Variation between two conditions (B compared to A) was calculated as a relative difference in lsmeans: (lsmeansB-lsmeansA)/lsmeansA. NEB ewes were compared to PEB ewes. Phenotype after the inflammatory challenge was compared to the phenotype before the inflammatory challenge. Significant effects were highlighted by stars: . P<0.1, * P < 0.05, **P < 0.01 and *** P < 0.001.

|  | Effect of DIET | |  | Effect of inflammatory challenge | | |
| --- | --- | --- | --- | --- | --- | --- |
| Trait | lsmeans  PEB | lsmeans NEB | Relative effect (NEB vs PEB) | lsmeans  Before | lsmeans  After | Relative effect  (After vs Before) |
| Weight Variation (%) | -1.1 | -2.64 | -139.8 . | -1.6 | -3.1 | -93.6 . |
| BCS | 30.5 | 28.78 | -5.6 * |  |  |  |
| Milk Variation (%) | 12.55 | -3.61 | -128.8 *** |  |  |  |
| Fat-to-Protein Ratio | 1.17 | 1.58 | +34.5 *** |  |  |  |
| Milk SCS |  |  |  | 4.09 | 9.74 | +138.4 *** |
| Log BHB (mmol/L) | -0.72 | -0.41 | +43.0 * |  |  |  |
| Log NEFA (mmol/L) | -1.09 | -0.02 | +97.7 *** |  |  |  |
| Glucose (g/L) | 0.71 | 0.67 | -6.4 . | 0.67 | 0.71 | +5.9 . |
| Log Insulin (µIU/mL) | |  |  |  |  |  |
| LOG T3 (ng/dL) | |  |  |  |  |  |
| Total blood leucocytes (10e9/L) | | |  | 7.32 | 5.21 | -28.7 *** |
| T lymphocytes (10e9/L) | |  |  | 2.71 | 1.8 | -33.8 *** |
| Monocytes (10e9/L) | |  |  | 0.22 | 0.14 | -33.1 * |
| Non T lymphocytes (10e9/L) | | |  | 2.68 | 2.04 | -23.8 ** |
| PMN (10e9/L) | |  |  | 1.53 | 1.05 | -31.3 ** |
| T lymphocytes (%) | |  |  |  |  |  |
| Monocytes (%) | |  |  |  |  |  |
| Non T lymphocytes (%) | |  |  |  |  |  |
| PMN (%) |  |  |  |  |  |  |


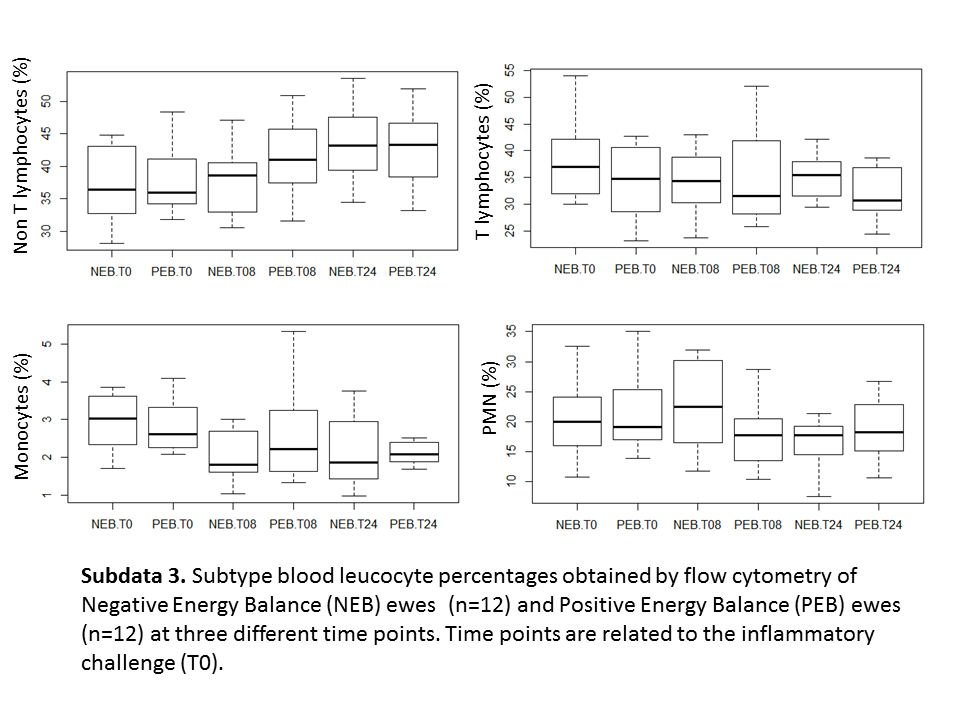


**Subdata 4.** Upstream transcriptional regulators estimated by Ingenuity from the 191 genes differentially expressed in response to energy restriction.

| **Upstream Regulator** | **Exp Log Ratio** | **Predicted Activation State** | **Activation z-score** | **p-value of overlap** |
| --- | --- | --- | --- | --- |
| PPARA | -0,328 | Activated | 2,563 | 3,21E-13 |
| PPARGC1B | 0,180 |  | -0,707 | 2,64E-09 |
| SREBF2 | -0,117 | Inhibited | -2,619 | 3,65E-08 |
| SIRT2 | 0,020 | Inhibited | -2,236 | 5,28E-08 |
| SREBF1 | -0,117 | Inhibited | -2,156 | 2,22E-07 |
| FOXO4 | 0,040 |  | 1,600 | 6,40E-07 |
| TP53 | -0,011 |  | 0,239 | 3,80E-06 |
| SIRT1 | 0,010 |  | 1,814 | 1,32E-04 |
| ESR1 | 0,329 |  | 1,451 | 1,35E-04 |
| KLF15 | -0,333 |  | 0,125 | 1,97E-04 |
| STAT2 | -0,085 |  |  | 1,97E-04 |
| RELA | 0,020 |  | -0,424 | 2,92E-04 |
| PPARD | 0,121 |  | 1,969 | 3,35E-04 |
| SP1 | -0,007 |  | -0,989 | 4,67E-04 |
| ESRRA | 0,059 |  | -0,447 | 5,52E-04 |
| ERG | 0,027 |  | 0,000 | 5,59E-04 |
| NR0B2 | 0,245 |  |  | 6,75E-04 |
| CREM | -0,008 |  |  | 7,40E-04 |
| SP3 | -0,004 |  |  | 7,73E-04 |
| USF2 | -0,003 |  |  | 8,81E-04 |
| RELB | -0,170 |  |  | 9,99E-04 |
| FOXN3 | 0,017 |  |  | 1,07E-03 |
| WT1 | -0,355 |  | 1,428 | 1,36E-03 |
| DEK | -0,030 |  |  | 1,41E-03 |
| USF1 | -0,025 |  |  | 2,26E-03 |
| NR3C1 | 0,056 |  |  | 2,96E-03 |
| CEBPA | 0,006 |  | 0,239 | 3,38E-03 |
| ESR2 | -0,457 |  |  | 3,86E-03 |
| HMGA1 | 0,030 |  | 1,387 | 4,00E-03 |
| PPARG | -0,025 |  | 0,810 | 4,46E-03 |
| thyroid hormone receptor |  |  |  | 5,83E-03 |
| CUX1 | 0,047 |  |  | 6,33E-03 |
| NFKB1 | -0,021 |  | -0,068 | 6,67E-03 |
| CCDC22 | -0,045 |  |  | 7,23E-03 |
| MBD4 | -0,068 |  |  | 7,23E-03 |
| BRCA1 | 0,022 |  | 0,277 | 7,79E-03 |
| LPIN1 | -0,017 |  |  | 9,97E-03 |
| PML | -0,118 |  | -1,964 | 1,07E-02 |
| EPAS1 | 0,221 |  |  | 1,12E-02 |
| HDAC3 | -0,027 |  |  | 1,17E-02 |
| AR | -0,037 |  | -1,254 | 1,18E-02 |
| NFKBIA | -0,081 |  | 0,239 | 1,23E-02 |
| HTT | -0,008 | Activated | 2,000 | 1,32E-02 |
| PRMT6 | -0,018 |  |  | 1,44E-02 |
| Hsf4 |  |  |  | 1,44E-02 |
| NF1 | -0,045 |  |  | 1,44E-02 |
| PREB | 0,026 |  |  | 1,44E-02 |
| ZEB2 | -0,076 |  |  | 1,51E-02 |
| ECSIT | -0,055 |  |  | 1,51E-02 |
| CREB1 | -0,004 |  | 1,067 | 1,51E-02 |
| RUNX1 | 0,019 |  |  | 1,56E-02 |
| GATA1 | -0,072 |  | 1,103 | 1,62E-02 |
| KDM5B | 0,013 |  | 1,000 | 1,68E-02 |
| CYLD | 0,033 |  |  | 1,74E-02 |
| CTBP1 | 0,038 |  |  | 1,74E-02 |
| NKX2-3 | -0,395 |  | 1,342 | 1,99E-02 |
| NR1I2 | -0,788 |  | -0,410 | 2,03E-02 |
| CNOT7 | 0,005 |  |  | 2,11E-02 |
| RXRG | 0,010 |  |  | 2,11E-02 |
| E2F4 | 0,008 |  |  | 2,33E-02 |
| IER3 | -0,028 |  |  | 2,86E-02 |
| COMMD1 | 0,008 |  |  | 2,86E-02 |
| PLAGL2 | -0,010 |  |  | 2,86E-02 |
| EFNA2 | -0,291 |  |  | 2,86E-02 |
| MYC | -0,082 |  | -0,036 | 3,04E-02 |
| NUPR1 | 0,005 |  | -0,841 | 3,06E-02 |
| ATF1 | 0,068 |  |  | 3,08E-02 |
| TFAP2A | -0,695 |  |  | 3,08E-02 |
| TFAP4 | 0,020 |  |  | 3,23E-02 |
| RARG | 0,138 |  |  | 3,39E-02 |
| DNMT3B | -0,127 |  |  | 3,43E-02 |
| NCOR2 | -0,000 |  |  | 3,54E-02 |
| MYBL2 | -0,107 |  |  | 3,54E-02 |
| HOXC6 | -0,125 |  |  | 3,54E-02 |
| CERS2 | 0,066 |  |  | 3,56E-02 |
| PPP1R13L | -0,052 |  |  | 3,56E-02 |
| E2F1 | 0,007 |  | 1,455 | 3,61E-02 |
| CDK2AP1 | -0,105 |  |  | 3,70E-02 |
| RXRA | -0,056 |  |  | 3,82E-02 |
| RXRB | -0,074 |  |  | 3,87E-02 |
| NKX2-1 |  |  |  | 3,92E-02 |
| MYOD1 | 0,704 |  |  | 4,06E-02 |
| DHX9 | -0,004 |  |  | 4,26E-02 |
| PSIP1 | 0,039 |  |  | 4,26E-02 |
| TAL1 | -0,149 |  |  | 4,56E-02 |
| RORA | 0,200 |  |  | 4,71E-02 |
| FOSL2 | 0,048 |  |  | 4,72E-02 |
| HIF1A | -0,092 |  | 1,291 | 4,94E-02 |
| BIRC2 | -0,053 |  |  | 4,95E-02 |
| PAX9 | 0,033 |  |  | 4,95E-02 |

**Subdata 5** : Primers for the qPCR analysis

| **PRIMER** | **SEQUENCE (5' > 3')** |
| --- | --- |
| PDK4_forward | TGTATCCCAAGCAAGGAACC |
| PDK4_reverse | TTTGATCCCTTAGCGTGTCC |
| BDH1_forward | TCACAGCTCCCAAGGAAAAC |
| BDH1_reverse | CAAAGGGCGCAGAGTAAAAC |
| CYP51A1_forward | CACGACTGTATGCGGATTTG |
| CYP51A1_reverse | TTCTTGATCTCCCGATGAGC |
| FADS1_forward | AGCAGCTTTCCCACTTTGAG |
| FADS1_reverse | ACGAACACGAGACCAAATCC |
| FDFT1_forward | AACTCATCACCAACGCACTG |
| FDFT1_reverse | AGGCAGCCAAAGTAGCAATG |
| HMGCS1_forward | ACAGCCGAGCATATTCCAAG |
| HMGCS1_reverse | AATGCTCCCCGTTACTGATG |
| MVD_forward | CGTCAAATACTGGGGAAAGC |
| MVD_reverse | CCGTCGTGGTGGTTTTTAAC |
| SQLE_forward | TGATGCTGTGATGGGAGTTC |
| SQLE_reverse | CAACAGTCAATGGAGCATGG |
| CPT1A_forward | CGCTGATGGTGAATAGCAAC |
| CPT1A_reverse | TGCGTCTGTAAAGCAGGATG |
| HPRPT_forward | TCCTCATGGACTAATTATGGACAG |
| HPRPT_reverse | CCACCCATCTCCTTCATCAC |
| GAPDH_forward | TGAGGACCAGGTTGTCTCCTG |
| GAPDH_reverse | CCCCAGCATCGAAGGTAGAA |
| YWHAZ_forward | GTCATCTTGGAGGGTCGTCT |
| YWHAZ_reverse | TCGAGCCATCTGCTGTTTTT |
| SDHA_forward | GTCAAGACTGGGGAGGTCA |
| SDHA_reverse | GTCGGTCTCGTTCAAAGTCC |
| RPL19_forward | CAACTCCCGCCAGCAGAT |
| RPL19_reverse | CCGGGAATGGACAGTCACA |


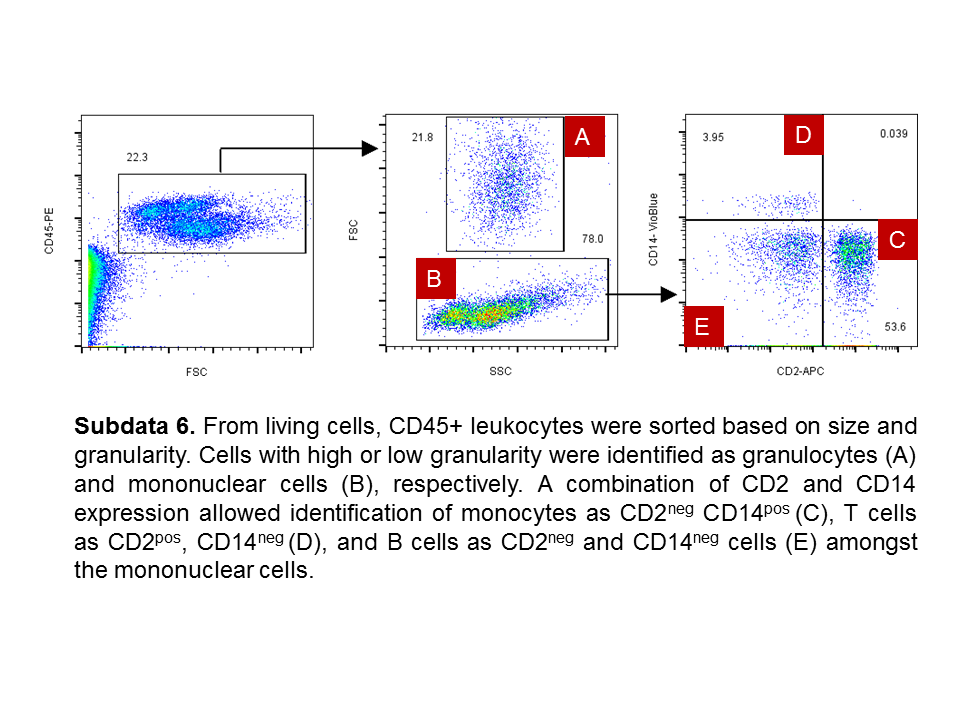

Supplement: Supplementary file 1 — Subdata figures [file 41598_2017_2391_MOESM1_ESM.doc]
